# Design of a cryptographically secure pseudo random number generator with grammatical evolution

**DOI:** 10.1038/s41598-022-11613-x

**Published:** 2022-05-21

**Authors:** Conor Ryan, Meghana Kshirsagar, Gauri Vaidya, Andrew Cunningham, R. Sivaraman

**Affiliations:** 1 Biocomputing and Developmental Systems Group, Lero, The Science Foundation Ireland Research Centre for Software, Computer Science and Information System Department, University of Limerick, Limerick, V94 T9PX Ireland UK; 2 Intel Research and Development Ireland Limited, Leixlip, W23 CX68 Ireland UK; 3 Department of Electronics Communication Engineering, School of Electrical and Electronics Engineering, Shanmugha Arts, Science, Technology and Research Academy, Deemed to be University, Thanjavur, 613401 India

**Keywords:** Computer science, Software

## Abstract

This work investigates the potential for using Grammatical Evolution (GE) to generate an initial seed for the construction of a pseudo-random number generator (PRNG) and cryptographically secure (CS) PRNG. We demonstrate the suitability of GE as an entropy source and show that the initial seeds exhibit an average entropy value of 7.940560934 for 8-bit entropy**,** which is close to the ideal value of 8. We then construct two random number generators, GE-PRNG and GE-CSPRNG, both of which employ these initial seeds. We use Monte Carlo simulations to establish the efficacy of the GE-PRNG using an experimental setup designed to estimate the value for *pi*, in which 100,000,000 random numbers were generated by our system. This returned the value of *pi* of 3.146564000, which is precise up to six decimal digits for the actual value of *pi*. We propose a new approach called *control_flow_incrementor* to generate cryptographically secure random numbers. The random numbers generated with CSPRNG meet the prescribed National Institute of Standards and Technology SP800-22 and the Diehard statistical test requirements. We also present a computational performance analysis of GE-CSPRNG demonstrating its potential to be used in industrial applications.

## Introduction

Random number generators^1^ are classified into two categories: true random number generators (TRNG)^2^ and pseudo random number generators (PRNG). TRNGs are able to generate randomness by relying on some physical source, such noise from thermal, atmospheric or radioactive decay sources. They are highly secure due to their reliance on such sources for strong entropy, but suffer due to their reliance on additional external devices. On the other hand, PRNGs generate random numbers deterministically based on an initial seed^3^ that, ideally, should be hard to predict and secure. If one gets hold of the seed or can influence the generation of the seed, one can predict the PRNG output and the whole system collapses. Thus, developing PRNGs that are secure enough still remains a key challenge to the researchers. The main advantages of using a PRNG are the rapidity and repeatability of output sequences^4^ with relatively small memory requirements. Designing and developing efficient PRNGs is always an area of interest to the researchers across all domains. Several approaches^5–7^ have been explored and investigated in the design of efficient PRNGs in the literature.

Most of the existing PRNGs are based on complex mathematical operations^8^ such as non-linear congruences^9^, linear feedback shift registers^10^ and quadratic residuosity, non-quadratic variants^11^ and cellular automata^12^, amongst others. PRNGs can be divided into two broad categories^13^, namely, basic PRNG and CSPRNG. Basic PRNGs are designed for simulations while CSPRNG are designed for cryptography. CSPRNG requirements fall into two groups: first, that they pass statistical randomness tests; and secondly, that they hold up well under serious attack, even when part of their initial or running state becomes available to an attacker. The random sequences generated by any CSPRNG are uniquely identified by the following three characteristics: (a) high entropy; (b) no repetition in strings generated; (c) zero correlation.

Grammatical Evolution (GE)^14–18^ is a bio-inspired population-based Machine Learning (ML) tool that makes use of Backus-Naur Form (BNF) grammars^19^ to generate legal structures for various problem domains. In this research work, we investigate GE as a potential entropy source to generate high entropy initial seed, as every evolutionary run is capable of producing a different seed. We propose a GE-based PRNG and GE-based CSPRNG, both of which can generate random sequences which adheres to meeting all standards of an excellent pseudo randomness.

## Results

### Generation of random sequences with *control_flow_incrementor*

The approach for generating random numbers for constructing CSPRNG is analogous to an incremental counter, where we define a loop to act as an incrementor counter and hence we name this approach control_flow_incrementor. This method uses a variable initialized to 0. To generate a new pseudo-random sequence, the current value of the variable is incremented and appended to the least significant bit (LSB) position of the initial seed. The resulting string is processed using a cryptographically secure hash algorithm such as SHA3-512^20^, depending on user requirement to make it secure against brute-force attacks. We can easily extend the size of the strings making it suitable for different applications with simple concatenation functions. For example, if we need a 1024-bit key, two 512-bit strings can be concatenated. As statistically inferred from our NIST experiments, we reseed our CSPRNG after generating every 4000 sequences for our 4096-bit CSPRNG to comply with the CSPRNG properties.

GE has the capability to generate n useful initial seeds depending upon the choices in production rules and the definition of production rule for pattern formation in the BNF grammar. We store all best individuals obtained during each run of GE in a repository. This can be useful in scenarios where we may not be able to obtain the best individuals in the first generation itself, given the inherent randomness of evolutionary computation. In such situations, we employ the mechanism of last in first out (LIFO) for seed extraction from the repository. We conducted an experiment to verify the potential of updating our entropy pool with unique seeds. In this experiment, 1,000 evolutionary runs resulted in 5076 unique seeds, in a time of 46.73 s, where each evolutionary run took an average time of 0.046 s.

### Statistical test for GE based CSPRNG

#### Randomness tests for GE-CSPRNG with NIST SP 800-22

GE-CSPRNG passed the statistical randomness test of output sequences from the National Institute of Standards and Technology NIST^21^ Special Publication (SP) 800-22 Statistical Test Suite. This comprises 15 tests, each of which generates a p-value in the range [0, 1], to demonstrate how well the generator holds up under serious attacks. Passing a particular threshold value, α, is indicative of success on a particular test.

In all 15 cases, α is defined to be 0.01, which indicates 99% probability of sequences to be random if the test is passed. The random sequences obtained by the *control_flow_incrementor* approach with GE were generated for 4096-bits as modern systems that use RSA^22^ for encryption standards usually require keys of 4096 bits. The longest data stream of 947,200 random bits passed all the 15 tests after which point the reseeding was done to obtain new samples. In both the cases, i.e. single objective CSPRNG which fulfils entropy requirements and the many objective CSPRNG which fulfils all three desired characteristics, viz, entropy, randomness and autocorrelation the difference in results is observed due to the fact of different seeds used to obtain the random sequences. The results of the tests are given in Table 1. The randomness and entropy of the strings are validated as all the tests are passed by the generated output sequences.

**Table 1:** Results from the NIST SP800-22 randomness test suite for GE-CSPRNG with a threshold of *α* = 0.01.

Test	Sequences obtained from seed with single objective fitness function		Sequences obtained from seed with many objective fitness function	
	p-value	Result	p-value	Result
Approximate entropy test (block = 8)	0.159423	Success	0.100883	Success
Block frequency test (block = 128)	0.471817	Success	0.336346	Success
Cumulative sums test (forward)	0.028936	Success	0.022369	Success
Cumulative sums test (reverse)	0.044248	Success	0.029855	Success
Fast Fourier Transform test (FFT)	0.584463	Success	0.081098	Success
Frequency test	0.026182	Success	0.017425	Success
Linear complexity (block = 500)	0.995472	Success	0.649274	Success
Longest runs of ones test	0.530367	Success	0.266024	Success
Non-overlapping templates test (block = 9, 000111101)	0.924066	Success	0.159012	Success
Overlapping template test of all ones test (block = 9)	0.670096	Success	0.659282	Success
Rank test	0.681196	Success	0.681668	Success
Runs test	0.461330	Success	0.092038	Success
Serial test 1 (block = 16)	0.331357	Success	0.559360	Success
Serial test 2 (block = 16)	0.570711	Success	0.742759	Success
Universal test	0.162531	Success	0.506254	Success

#### Randomness tests for GE-CSPRNG with diehard battery of tests

Diehard^23^ is the statistical test suite developed by George Marsaglia for estimating the statistical independence of random numbers through which the randomness has been confirmed. It requires 80 million bits for conducting the 18 tests to assess the quality of the CSPRNG. For Diehard tests, we generated 80 million bits with *control_flow_incrementor* approach and the results have been tabulated in Table 2.

**Table 2:** Results from the diehard battery of tests for GE-CSPRNG with a threshold of *α* = 0.01.

Test	Single objective		Many objectives	
	p-value	Result	p-value	Result
Birthday spacing	0.7466	Success	0.2564	Success
Overlapping 5-permutation	0.5467	Success	0.5346	Success
32 × 32 binary matrix	0.9848	Success	0.7977	Success
6 × 8 binary matrix	0.1799	Success	0.8600	Success
Overlapping pairs sparse occupancy	0.8238	Success	0.9221	Success
Overlapping quadruples sparse occupancy	0.9718	Success	0.9858	Success
DNA	0.9219	Success	0.9756	Success
Count the 1's test on a stream of bytes	0.5107	Success	0.5401	Success
Count the 1's test for specific bytes	0.9567	Success	0.9878	Success
Parking	0.3296	Success	0.6486	Success
Minimum distance	0.7342	Success	0.4018	Success
3D spheres	0.5165	Success	0.6312	Success
Squeeze	0.0647	Success	0.9935	Success
Overlapping sum	0.2822	Success	0.1674	Success
Runs	0.5303	Success	0.8222	Success
Craps	0.1457	Success	0.2928	Success
Bitstream	0.9255	Success	0.9479	Success

The 2-D spheres test was integrated with 3-D spheres and thus we tested our random sequences on 17 tests. On observing the results, out of 17 tests, all tests have been passed to meet out the diehard criteria, which ensures the randomness of the proposed PRNG.

#### Security analysis of GE-CSPRNG

Reverse engineering is a crucial attack that exploits the inherent features of any design. It helps the attacker gain knowledge about the design and to analyse the weaker points of it. It leads to cloning of the design/device, fault injections, trojans, cryptanalysis etc. It has three fundamental phases namely information extraction, modelling and analysis. The proposed PRNG integrates a novel random seed generation with a SHA3-512 to achieve adequate randomness. As the proposed BNF grammar for GE-PRNG is flexible, the production rules can be extended by merely adding additional choices for symbols, thus having the potential to generate ∑2^n^ number of unique seeds, where n is the total number of choices for each of the production rules. The BNF grammar proposed in the article has the potential to generate 1,514,240 (as explained in Eq. (1)) unique seeds with the permutations of the production rules and available choices. Hashing the initial seed with SHA3-512 ensures it against session replay attacks and brute force attacks.

1 $$\begin{array}{l}Permutation\left(\mathrm{BNFGrammar}\right)=ch\left(1\right)\times ch\left(2\right)\dots \times ch\left(8\right)\end{array}$$

$$\mathrm{where\, ch}(1)\mathrm{ is\, the\, number\, of\, choices\, for\, rule }1,\mathrm{ ch}(2)\mathrm{ is\, the\, number \,of\, choices\, for\, rule }2,\mathrm{ \, etc}.$$

The number of choices for each of the eight rules in this grammar are 1, 1, 1, 8, 26, 26, 28 and 10. This gives the possible permutations for the grammar as 1,514,240, which equates to the number of unique seeds for our grammar. Similarly, with this logic, we can generate a larger number of unique initial seeds simply by adding more combinations of choices in the BNF grammar by the inclusion of additional production rules. This feature shows the flexibility of GE-PRNG to be scalable for applications that cater to a large number of users needing unique initial seeds for generating random numbers.

The length of random numbers generated by a PRNG after which the sequences start to repeat themselves is called the period of the PRNG. The period of the proposed PRNG depends upon the limit of variables for the incrementor loop in the system program. For example, if we use long long int in C, the period for the PRNG would be 9,223,372,036,854,775,807, as that is the limit for a long long int in C.

Also, the proposed CSPRNG meets the Avalanche criteria in which the PRNG produces a new set of random values even for a single bit change in the inputs. Since the SHA3-512 has a strong foundation of irreversibility, it helps the PRNG to produce high quality irreversible random numbers. Hence, the problem of reverse engineering can be alleviated.

#### Analysis of computational performance of GE-CSPRNG

To analyse the Encryption throughput (ET) and Number of cycles required for encrypting per byte (NpCB) for GE-CSPRNG we followed the same procedure as used by the authors^24^. By using the functionality available in Python’s OpenCV library, we generated 10 plain RGB images of varying sizes. We then encrypted the images, by using the encryption method^25^, which is basic shuffling of pixels in the RGB images. The encryption was done with the keys obtained from GE-CSPRNG and using Python’s *rand()* function for comparative analysis. Then we calculate ET and NPCB using Eqs. (2) and (3).

2 $$\large \large \begin{array}{c}ET=\frac{\mathrm{Generated\, data\, size }}{\mathrm{Average\, generation\, time}}\end{array}$$

3 $$\large \large \begin{array}{c}NpCB=\frac{\mathrm{CPU \, main\, clock \,frequency }(\mathrm{Hz}) }{\mathrm{ET }(\mathrm{bytes}/\mathrm{s})}\end{array}$$

Table 3 shows the comparative analysis of ET for ten RGB images with GE-CSPRNG and the Python *rand()* function, that is Mersenne Twister (MT). The comparative ET from both the approaches show that GE based PRNG is performing well in terms of speed and surpassing the one with Python’s *rand()* function. The best ET and NpCB for GE-PRNG was found for the image with size 100 × 100, followed by 800 × 800.

**Table 3:** Speed analysis of GE based PRNG and Python based PRNG with encryption of RGB images.

Image size (pixels)	Image size (Kbs)	GE-based PRNG				Python based PRNG		
		Time(s)	ET (Kb/s)	ET (Mb/s)	NpCB	Time(s)	ET (Kb/s)	NpCB
100 × 100	30	0.023644	**1268.809**	**1.26880963**	**788.140296**	0.036765	815.9859	1225.5112
200 × 200	118	0.109337	1079.228	1.07922884	926.587541	0.136072	867.1823	1153.1599
300 × 300	264	0.266003	992.4686	0.99246862	1007.58852	0.279992	942.8829	1060.5770
400 × 400	469	0.482753	971.5112	0.97151123	1029.32417	0.507970	923.2818	1083.0929
500 × 500	733	0.796582	920.1812	0.92018121	1086.74245	0.800740	915.4021	1092.4160
600 × 600	1055	1.087610	970.0166	0.97001660	1030.91018	1.198891	879.9797	1136.3897
700 × 700	1436	1.555533	923.1561	0.92315614	1083.24037	1.590063	903.1088	1107.2862
800 × 800	1876	1.539821	**1218.322**	**1.21832274**	**820.800566**	2.035266	921.7467	1084.8966
900 × 900	2374	2.507421	946.7893	0.94678936	1056.20113	2.584014	918.7255	1088.4643
1000 × 1000	2930	3.223342	908.9942	0.90899420	1100.11702	3.420089	856.7028	1167.26

The experiments were performed by employing Linux version 16.04, the GE tool, libGE-version 0.32 in C/C++ from Biocomputing and Developmental Systems Research Group at University of Limerick, and Python 3.7 with the following configurations: Intel(R) Core (TM), i5-1035G1 CPU 1 GHz 8 GB RAM.

#### Simulation based tests for GE based PRNG

Random numbers find their applicability in various simulations and for validating ML models. We evaluated the potential of GE based PRNG for usage in simulations and sampling applications with two different methods, viz, Monte Carlo and fitting ML regression models.

#### Monte Carlo simulation for the estimation of pi

Monte Carlo^26^ simulations are widely used for ensuring the quality of random numbers generated. They are used to validate whether a given functionality of PRNG successfully achieves its target goal by calculating the value of pi from the random numbers generated by the PRNG. The value of pi is estimated^27^ with the help of Eq. (4) for calculating the radius of a circle.

4 $$\mathrm{r}=\sqrt{\left({\mathrm{x}}^{2}+{\mathrm{y}}^{2}\right)}$$

5 $$\mathrm{pi}=4\times \frac{\mathrm{N}\left(\mathrm{points\, inside\, the\, circle}\right)}{\mathrm{N}\left(\mathrm{total\, points}\right)}$$

where x and y are random numbers generated by GE-PRNG. 1,000,000 or more random numbers are generated in the range from 0 to 1 and the value for Eq. (5) is calculated. If the value of the equation is less than 1, the point is placed inside the circle, i.e., the random numbers pair is valid. If the value of the equation is greater than 1, the point is placed outside the circle and the point is discarded while calculating the value of pi. At the end of the prescribed 1,000,000 runs, the value is obtained by Eq. (5) and then it is compared with the actual value of *pi*. The closer the estimated value to the actual value of *pi*, the better is the performance of the PRNG. The value of *pi* noted with our observation over 1,000,000 runs is 3.146564000, while the actual value of *pi* is 3.1415926535^28^ up to 6 decimal precision which gives a strong validation of randomness.

#### Coverage analysis

To validate the suitability of GE-PRNG for its potential use of generating samples for ML datasets, we generated samples across 16 real-world benchmark regression datasets with varying numbers of instances. The test evaluates the quality of random samples generated in terms of mean and standard deviation with respect to ground truth (entire data). The samples were generated for columns of varying lengths representing different data types such as categorical, integers and float. The samples were visualised by plotting histograms to analyse their distribution. A comparative analysis was then performed against the samples obtained from Python’s *rand()* function-based MT. We observed that the samples from GE-PRNG followed the same type of data distribution^29^ as observed in the original datasets. This implied that in future GEPRNG can play a crucial role in synthetic data generation. The results of the analysis are presented in Supplementary Table S1.

## Discussion

There have been several PRNGs proposed in the literature that incorporate bio-inspired methods, such as GP and Genetic Algorithms (GA)^30^. For example, Koza^31^ used GA to transform a seed **J** into a PRNG using a set of standard arithmetic operators and a parameter set of {population size: 500, crossover: 0.9 and mutation: 0}. The resulting PRNG has been tested digitally on software and passed both Gap-Measure and Chi-square tests. Poorghanand^32^ employed a GA using 16 Linear Feedback Shift Registers with XOR and inverse-XOR gates to generate high entropy 128-bit random numbers and successfully passed the NIST suite of tests. A Hebbian neural network initialised by a GA was proposed by Jhanjharia^33^. With a parameter set of {population size: 50, crossover: 0. and mutation: 0.05} this was capable of producing 192-bit output and successfully passed each of the Cumulative Frequency, Gap-Measure and Chi-square tests. More recent work by Kosemen^34^, extended Koza’s work and resulted in a design that passed the NIST suite of tests, producing output within 0.24960 s. This used the following parameters: {population size: 50, crossover: 0 and mutation: 0.5}. Due to GE’s modular structure, it is highly convenient as an alternative search strategy, whether evolutionary, deterministic or any other. Such a flexible approach to Genetic Programming (GP) makes GE a robust machine learning tool that can be applied to a diverse set of problem domains. Moreover, GE’s potential as an entropy source for a secure CSPRNG, using the basic BNF grammar, makes it possible to arm GE with problem-specific constructs as demonstrated with our approach. Further, GE-PRNG holds the promise of a potential rich source for generating synthetic datasets for fitting ML regression models. Due to the ability to customize the production rules of BNF grammar, GE-PRNG can serve applications requiring one-time password (OTP) generation and the keys obtained with GE-CSPRNG can be employed for storage encryption, network encryption, confidential compute, etc. We illustrate in Supplementary Table S2, a comparative analysis of GE-based CSPRNG with respect to existing software and hardware.

## Conclusion

We have presented a combined application of GE and our novel approach, *control_flow_incrementor*, for the design of a basic and cryptographically secure pseudo random number generator. We used *control_flow_incrementor* to generate random numbers and GE as the entropy source to return an initial seed. The validations of the CSPRNG with NIST SP800-22 and Diehard battery of tests indicate the feasibility of GE as a source of initial seeds leading to the efficient construction of CSPRNG. By utilizing the seed repository for reseeding the initial seed, our CSPRNG is able to generate highly uncorrelated random sequences at a faster rate with minimal computational costs, making it highly efficient for securing sensitive data. Monte Carlo simulations were performed to validate the quality of random numbers for sampling with GE-PRNG. Furthermore, extending production rules with additional choices will make it adaptable across a wide range of industrial applications.

## Methods

This section discusses the procedure of obtaining initial seeds for the design of PRNG and CSPRNG with GE. The functionality of GE is based upon the generation of structures described by a formal language grammar, typically in the form of a BNF.

**Figure 1:**
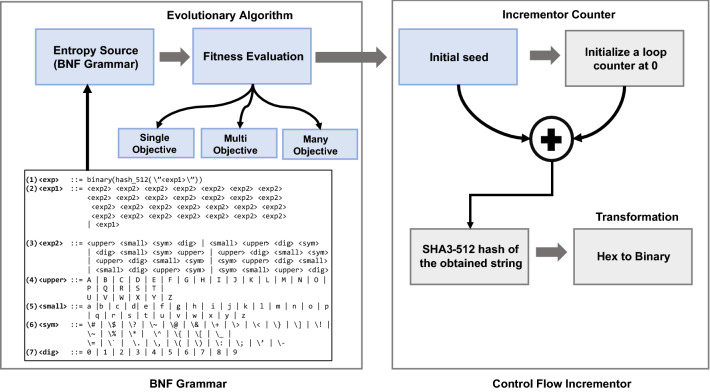
Pipeline for the design of GE-PRNG with the *control_flow_incrementor* approach. The process starts with using BNF grammar as entropy source to obtain the initial seed and subsequently, uses the seed as an input to the *control_flow_incrementor* function which generates random numbers.

At its heart is a GA that searches through the space of syntactically legal structures. The GA individuals, or *genomes*, are variable length binary strings that get mapped onto a *phenotype*, the actual structure being created, which is typically a program or some other, complex structure. The mapping process is guided by the grammar, which, in BNF can be represented as the tuple {S, N, T, P}, where **S** is a start symbol, **T** is the set of *terminals*, that is, items which can appear in the language. **N** is the set of *non-terminals*, temporary items to facilitate the mapping process, and **P**, a set of production rules that expand **S** into a legal structure consisting of only terminals. Figure 1 shows a set of production rules that map the start symbol < expr > to a seed, which is made up entirely from terminals.

GE operates by evolving populations of these individuals, each of which is tested for *fitness*, i.e. how well they perform at the task in hand, entropy in this case. These individuals are then probabilistically selected for crossover and mutation, based on their fitness, and a new population is created.

As shown in Fig. 1, the GE-based PRNG takes a 512-bit initial seed and, combined with the proposed approach, *control_flow_incrementor*, generates random numbers. Our *control_flow_incrementor* follows the logic of an incrementor counter initialized at 0. With each iteration, the value of the counter is incremented by any pre-decided number (we have used 1, although this can be any value) and appended to the initial seed obtained from the entropy source. This intermediate string is then hashed with SHA3_512 to generate secure random numbers.

The evolutionary process that leads to initial seeds with GE can be summarized in the following steps:

- 1. Initialise evolutionary parameters {population, generations, crossover, mutation};
- 2. Obtain binary string from BNF grammar;
- 3. Evaluate the individuals with a fitness function^35^ using Shannon’s entropy^36^ equation (7) to get the fitness scores;
- 4. Generate new population for next generation using crossover and mutation;
- 5. Repeat until there is no improvement in fitness.

We fulfill all the requirements of a desirable PRNG by mapping each of characteristics with a fitness function as seen in Table 4. We use the Shannon’s entropy as a fitness function F1 to obtain high entropy initial seeds. The fitness function Hamming Distance, *F2*, ensures that unique seeds are generated with each evolutionary run. These seeds are subsequently stored in a repository to reseed the PRNG after a defined period. Period of a PRNG is the number of pseudorandom sequences after which the PRNG needs to be reseeded to avoid repetition in the output sequences, thus maintaining randomness. Moreover, these seeds from the repository can also be used as an entropy source thus avoiding evolutionary runs for seed generation. Similarly, the fitness function, *F3*, Autocorrelation, ensures no repetition of patterns within the same seed.

**Table 4:** The different fitness functions along with their range of values used for the design of GE-PRNG.

Fitness function	Range	Objective function	Remarks
F1: Shannon’s entropy	[0–8]	Maximise	This fitness function helps to obtain an initial seed with optimal entropy
F2: Hamming distance	[0–512]	Maximise	The usage of this fitness function ensures generation of unique seeds
F3: Autocorrelation	[0–1]	Minimise	The functionality of the fitness function is to have high randomness within the initial seed

A fitness function, in the context of evolutionary computation, is the measure of how good a solution is for a given problem. The fitness function, *F1*, defined in Eq. (7) is based upon Shannon’s entropy function as defined in Eq. (6).

6 $$\mathrm{H}\left(\mathrm{X}\right)=-{\sum }_{\mathrm{i}=1}^{\mathrm{N}}{\mathrm{p}}_{\mathrm{i}}{\mathrm{log}}_{2}{\mathrm{p}}_{\mathrm{i}}$$

7 $$\mathrm{fitness}= \sum_{\mathrm{n}=1}^{8}\left(\frac{-{\sum }_{\mathrm{i}=1}^{\mathrm{N}}{\mathrm{p}}_{\mathrm{i}}{\mathrm{log}}_{2}{\mathrm{p}}_{\mathrm{i}}}{\mathrm{n}}\right)$$

where $$\mathrm{N\, is \, the \, total\, number\, of\, permutations \, possible\, in\, the\, binary\, sequence\, of \, the\, initial\, seed }(\mathrm{e}.\mathrm{g}.\mathrm{ for}\, 1 \, \mathrm{ bit},\, \mathrm{ N \, is }\, 2\, \left\{\mathrm{0,1}\right\} \mathrm{and \, for\, }2\, \mathrm{ bit},\, \mathrm{ N \, is\, }4\, \{\mathrm{00,01,10,11}\})$$. $$\mathrm{pi \, is \, the\, frequency \, of \, the \, patterns\, of \, }0\, \mathrm{s \, and\, }1\mathrm{s \, in \, the\, binary\, sequence}.$$ $$\mathrm{n \, is \, the \, number\, of\, bits\, in\, the \, range }[1-8]$$.

The fitness function in Eq. (7) is the summation of all the *n*-bit entropy values H_1_…..H_8_, divided by *n*, i.e. number of bits^30^. The division by *n* ensures equal contribution of all *n*-bits entropies in the final calculation of fitness. The sample example of an initial seed returned from GE, below, depicts the procedure followed to calculate the entropy.

10011101011011001001100011100110111111111110110010010001100011111010000011000010010111111010110101001110000111101001111000010100100010010000101101010001101110100001001010001101110011111100001111101101010100010011110101000001001110010011100100011010110100101101010011110011110100010000000101100111000011011000011000001110111110000000110100101100101100100100110001111010110010010000001000000011110101011000111110010111101001011100110110011001111001110001111100000001010111110001011101110100000010100010000100111110

All 1-bit possible combinations with 0s and 1s are {0,1}. Initially, we calculate the frequencies of these combinations, which, in this case, are 260 and 252 respectively. Then we calculate the value H1 with Eq. (6), which is 0.9998. Then we calculate the value of H_1_ /*n*, where *n* is 1 in this case. This gives the entropy as 0.0999.

Similarly, all the 2-bit possible characters are {00, 01, 10, and 11} and their respective frequencies are 137, 122, 123, 129 respectively. The value of H2 is 1.99. The final entropy for 2-bit is *H*-value/*n*, i.e 1.99/2, which gives final entropy as 0.995. In this way, we calculate all the *n*-bit entropy values up to 8, and the value for each *n*-bit entropy lies in the range [0-1]. This binary notation of the initial seed obtained from GE has a final fitness value (entropy) of 7.962 as shown in Table 5.

**Table 5:** Sample fitness score calculation.

#bits (n)	*H*-value	Entropy (*H*-value/n)
1	H_1_ = 0.998	0.998
2	H_2_ = 1.99	0.995
3	H_3_ = 2.99	0.996
4	H_4_ = 3.99	0.997
5	H_5_ = 4.97	0.998
6	H_6_ = 5.98	0.996
7	H_7_ = 6.85	0.994
8	H_8_ = 7.91	0.988
∑Entropy		7.962

The entropy of each H value is summed.

### Single objective fitness function

**Figure 2:**
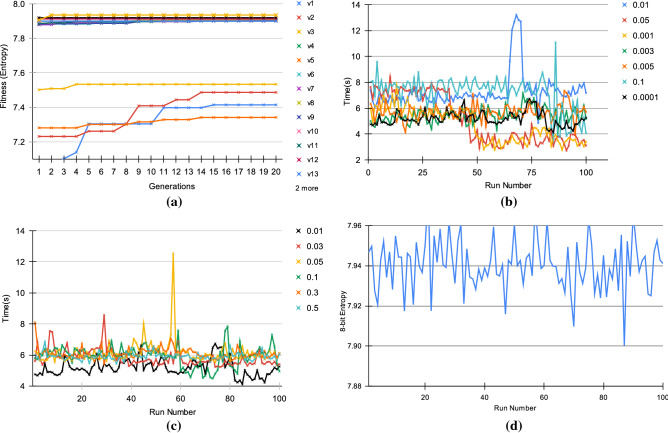
Tuning of BNF grammar and genetic operators, mutation and crossover, to obtain the optimal entropy. In all combinations of the BNF Grammar as depicted in (**a**) where 20 generations were sufficient to obtain the optimal entropy. The initial seed leading to optimal entropy using the 15th version of BNF Grammar was secured with SHA3-512, whereas in the previous 14 versions, the hashing algorithm SHA2-512 was used to secure the initial seed. In all of the cases, the entropy remained in the range 7.92 to 7.94. (**b**) Values for crossover were varied from 0.0001 to 0.1 while keeping mutation fixed at 0.01. With the value of 0.0001, the optimal entropy remained consistent and the time taken to obtain the entropy was also consistent for that value. Similarly, in (**c**), we vary the mutation rate from 0.01 to 0.5 while keeping crossover fixed at 0.001. In this case, the most consistent value for time taken was exhibited by a mutation rate of 0.01. Finally, these two values were used with the 15th BNF grammar version to produce an optimal entropy value of 7.940560934 from 100 runs as illustrated in (**d**).

To generate high entropy initial seeds, we first performed preliminary experiments with GE on a variety of different BNF grammars with fitness function *F1*. We obtained an entropy of 7.31 in version 1 with the evolutionary parameters {population size: 10, generations: 15, crossover: 0.9, mutation: 0.04} and subsequent modifications to the production rules and evolutionary parameters led to the optimal parameters for our fifteenth version as seen in Fig. 2a and details of entropy improvements depicted in Supplementary Table S3.

The optimal evolutionary parameters obtained were as follows: {population size: 2, generations: 5, crossover: 0.0001, mutation: 0.01}. The initial seed obtained from GE is subsequently used to generate random sequences with the *control_flow_incrementor* approach. This strongly supports the use of GE as a high-quality entropy source for initial seeds which is independent of hardware or software requirements. The production rules in grammar can be used to yield arbitrarily large output random sequences, thus making it easily adaptable across diverse applications. Hence, we propose GE based PRNG to be suitable for domains such as sampling Machine Learning (ML) models, generating One-time Passwords (OTP), and to be used as a CSPRNG. The genetic operators, mutation and crossover, were derived after performing a series of experiments over 100 runs. Figure 2b,c show the tuning of crossover and mutation rates, while Fig. 2d shows the mean entropy of the PRNG with the fifteenth version of grammar. The values for the genetic operators of crossover and mutation were varied in the range [0,1] and experimented on all the fifteen versions of the BNF grammar which resulted in the final values of crossover fixed at 0.0001 and mutation at 0.01. This strongly validates the potential of GE-PRNG as being computationally efficient in terms of speedup and complexity.

### Multi-objective and many objective fitness functions

**Figure 3:**
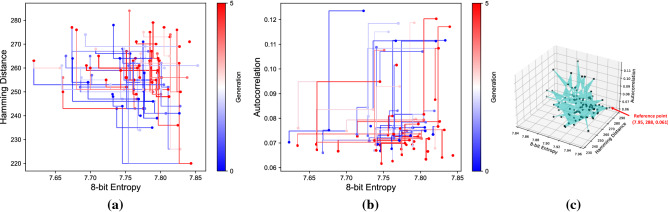
Results of the fitness evaluations with multi-objective and many-objective fitness functions for a 512-bit seed generated by GE-PRNG. (**a**) The Pareto fronts for a multi-objective fitness function made up of F1, entropy, and F2, Hamming distance. This indicates the maximum dissimilarity between any pair of strings generated by GE-PRNG while maintaining the optimal achieved entropy for a particular generation; (**b**) shows the Pareto fronts for a multi-objective fitness function made up of F1, entropy, and F3, Autocorrelation. This experiment validates minimal autocorrelation within a string without impacting the optimal achieved entropy. Finally, (**c**) shows the hypervolume for a many-objective fitness function made up of all three, F1, F2, F3. The combination of all three objectives produces a solution that meets all the prescribed characteristics of an PRNG.

The subsequent series of experiments involved the combinations of fitness functions in a pair of two to achieve the desirable characteristics of PRNGs. For two objectives, we use NSGA-II in two setups: (a) *F1, 8-bit entropy* and *F2, Hamming Distance* and (b) *F1, 8-bit entropy* and *F3, Autocorrelation*. Figure 3a,b illustrate the results obtained for two objectives after performing 30 revolutionary runs. The optimal solutions lie in the region of [7.65, 7.95] for *F1* and for *F2*, the region is [220, 280]. Similarly, the region for *F1* and *F3* is [7.65, 7.95] and [0, 0.09] respectively.

To achieve all the characteristics of a CSPRNG, we used the combination of all the three fitness functions as a single setup combining *F1*, *F2* and *F3*. For experimentation purposes, we used NSGA-II with pymoo^37^ to achieve many-objective fitness functions. The seed obtained from a many-objective fitness function will be the one with the maximum entropy, the maximum hamming distance and the minimum autocorrelation. The optimal solution obtained for three objectives was evaluated with a hypervolume indicator from a reference point [7.95, 298, 0.06]. The hypervolume indicator^38^ is a measure for many-objective optimization where the volume enclosed between a reference point and a Pareto front is calculated. A reference point^39^ is the maximum/minimum point obtained in the series or runs for an evolutionary experiment. The smaller the volume, the more optimal are the solutions obtained from the runs. Figure 3c illustrates the hypervolume obtained after combining *F1*, *F2* and *F3* over 30 runs. Unlike multi-objective experiments, the individuals here lie in the average region {[7.60, 7.96], [230, 290], [0.06, 0.11]}.

## Supplementary Information

Supplementary Tables.

## Data Availability

All data generated or analysed during this study are included in this published article and its Supplementary Information file. The tool libGE - version 0.32 used in the experimentation will be made available upon request to the corresponding authors.
