# Supplementary material for: Design of a cryptographically secure pseudo random number generator with grammatical evolution
Source: Sci Rep. 2022 May 21;12:8602. doi: 10.1038/s41598-022-11613-x (PMC9124193; doi:10.1038/s41598-022-11613-x)
Supplement: Supplementary file 1 — Supplementary Tables. [file 41598_2022_11613_MOESM1_ESM.pdf]

Supplementary Information

Supplementary Table S1: Comparative analysis of GE-PRNG vs Python rand () for validating random samples with regression datasets [1] for coverage analysis

Dataset	# Instances	Name of sampled column	Histogram
Canada (per capita income)	100	Per Capita Income	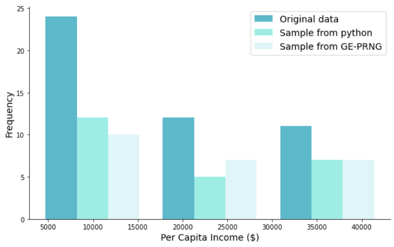
Cancer	3048	Average deaths per year	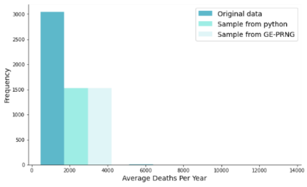
Car Prices	4341	Type of sellers	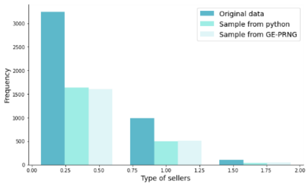
Covid-19 Test Cases	41706	Country	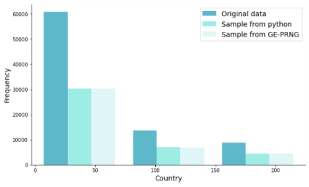
Crime	46470	Number of offenses	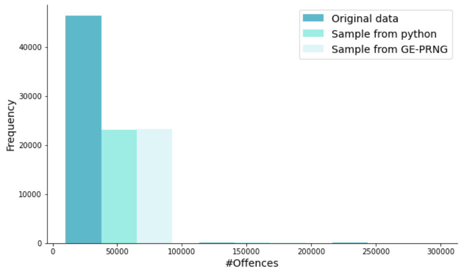
Airlines Delay	1048575	Aircraft Delay	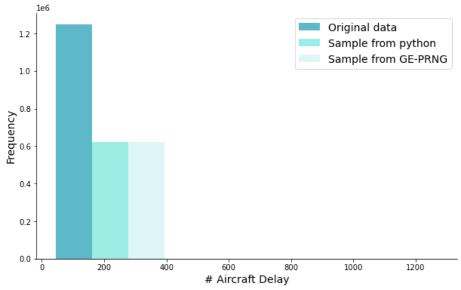

Fish Market	160	Type of species	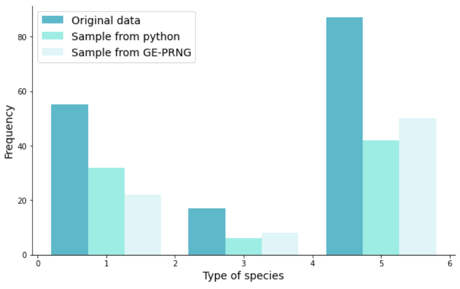
German Credit Cards	1000	Age	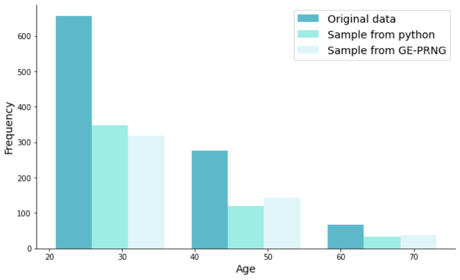
Medical Costs	53393	Age	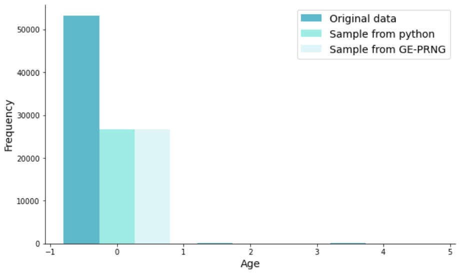
House Prices	85905	Price of Unit Area	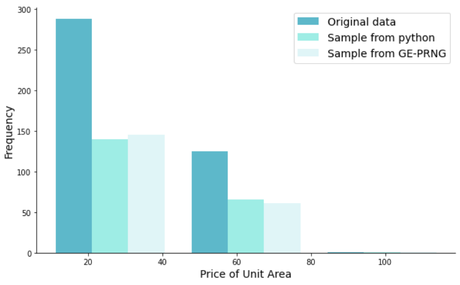
Insurance costs	1339	Region	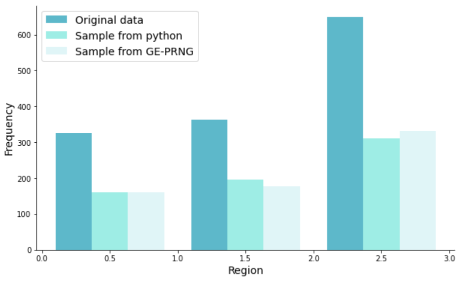
Salary of Employees	36	Years of experience	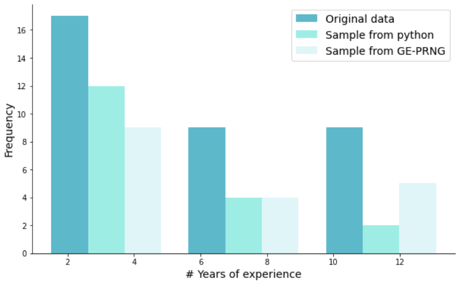

Weather prediction	119041	Mean Temperature	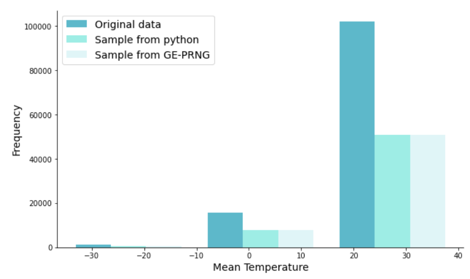
Weather prediction	162	Elevation	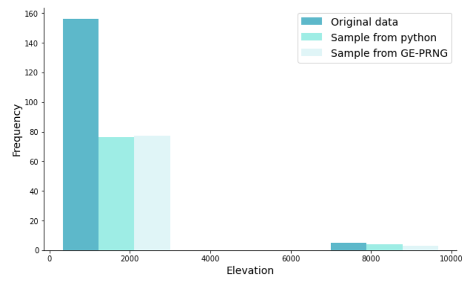
Red Wine	1600	Quality	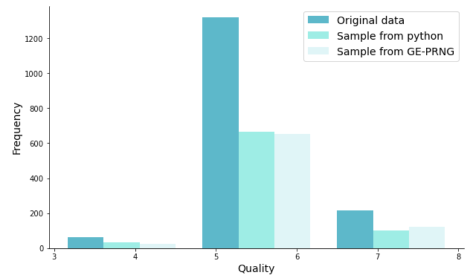
Zoo	102	Animal Type	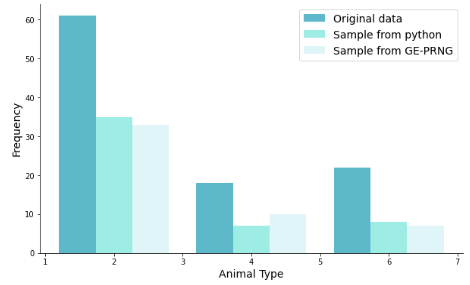

Supplementary Table S2: GE-PRNG vs existing PRNGs

Features	Software PRNGs					Hardware PRNGs					GEPRNG
	**Linux (/dev/urandom) [2]**	**Python [3]**	**AES [4,5]**	**Blum-Blum Shub [6]**	**ChaChaRNG [7]**	**ReRAM Based PRNG [8]**	**1-D Pseudo-Chaotic [9]**	**PRNG Based on Newton Complex Maps [10]**	**Fractal Based PRNG [11]**	**Micro computer based PRNG [12]**	
Source of randomness	Operating system API	Random library in Python	Key from any API	API/ System default random library	NA	Resistive Random-Access Memory (ReRAM) with Linear Feedback Shift Register (LFSR)	Arnold Cat chaotic map	Newton Raphson Method	Fractals (Chaos game)	Chaotic system with RK4 method	BNF Grammar
Width of random bits	*Variable:* [10, max range of system]	- *Variable* - 64-bits basic version	-*Variable*: 128, 192, 256	NA	NA	14	- *Variable:* 32, 48, 64, 80. - Maximum is 80 bits.	49	8	8	-*Variable* 256, 512, 1024, 2048, 4096
Scheme of statistical evaluation	NA	NA	NIST FIPS 142-2	NA	TESTU01 and NIST SP 800-22	NIST SP 800 – 22	- NIST SP 800 – 22 - Lyapunov exponent calculation for ensuring the chaoticity.	NIST SP 800 - 22	NIST SP 800 - 22	NIST SP 800 - 22	-NIST SP 800-22 -Monte Carlo Simulations -Dataset sampling for regression analysis -Linear complexity analysis
Key space	NA	2^19937 - 1	2^56	NA	NA	2^14	2^80	2^588 (2nd order)	2^8	2^8	**2 ^ 128**
Availability of reseeding	Yes	Yes	No	NA	NA	Available on demand. RTN is a control signal has been used to trigger the reseeding.	NA	NA	NA	NA	Yes
Throughput	NA	26.13 billion random samples per second	139 MiB/s for 128-bits version	NA	NA	Variable (Mbps to Gbps depends on the system clock)	NA	2.9 Mbps (2nd order)	NA	NA	40.322 Mbps
Remarks/Limitations	-Reseeding requires more time if the entropy pool does not contain randomness	Only CryptMT variant is secure	NA	NA	NA	- On demand reseeding -ReRAM is a costly product	- Quantization issues in float to binary conversion - No Reseeding	- Quantization effects will occur since IEEE 754 double precision was adopted.	- Use of 3D chaotic model for key generation.	- Only 8 bits extracted from a 32 bit	
OS compatible	Linux, Solaris, MacOs, FreeBSD	All systems with Python support	All systems that use AES encryption	Yes	OpenBSD/FreeBSD/NetBSD	NA	NA	NA	NA	NA	

The following table shows the optimal entropy obtained with version 15 of BNF Grammar. The evolutionary parameters like population and generation varied between [1-20] across the versions. The time taken by the initial versions was much higher as 10s which reduced to 5s in the intermediate versions and finally reached to 1s in the 15th version.

Supplementary Table S3: BNF Grammar versions

<table>
<tr><th>#Version</th><th>BNF Grammar</th><th>Average Entropy</th></tr>
<tr><td>1</td><td><exp>::=\"<exp1>\";
<exp1>::=<upper><small><sym><dig>|<exp1><exp1>
<upper>::=A|B|C|D|E|F|G|H|I|J|K|L|M|N|O|P|Q|R|S|T|U|V|W|X|Y|Z
<small>::=a|b|c|d|e|f|g|h|i|j|k|l|m|n|o|p|q|r|s|t|u|v|w|x|y|z
<sym>::=\#|\$|\?|\~|\@|\&|\/|\.|\+|\>|\<
<dig>::=0|1|2|3|4|5|6|7|8|9</td><td>7.30</td></tr>
<tr><td>15</td><td><exp>::= binary(hash_512(\"<exp1>\"))
<exp1>::=<exp2><exp2><exp2><exp2><exp2><exp2><exp2><exp2>|<exp1>
<exp2>::=<upper><small><sym><dig>|<small><upper><dig><sym>|<dig><small><sym><upper>|<upper><dig><small><sym>|<upper><dig><small><sym>|<sym><upper><dig><small>|<small><dig><upper><sym>|<sym><small><upper><dig>
<upper>::=A|B|C|D|E|F|G|H|I|J|K|L|M|N|O|P|Q|R|S|T|U|V|W|X|Y|Z
<small>::=a|b|c|d|e|f|g|h|i|j|k|l|m|n|o|p|q|r|s|t|u|v|w|x|y|z
<sym>::=!|"#"|$|'%'|&|\(|\)|*|+|,|-|.|\/|:|;|"<"|=|">"|?|@|\[|\\|\]|^|_|"`"|{|}|~|"|"|'\'"|"'"
<dig>::=0|1|2|3|4|5|6|7|8|9</td><td>7.94</td></tr>
</table>

References

1. Rachael Tatman. Datasets for regression analysis retrieved from https://www.kaggle.com/rtatman/datasets-for-regression-analysis
2. Lloyd, Jack (2008-12-09). "On Syllable's /dev/random". Retrieved 21-11-2021
3. https://docs.Python.org/3/library/random.html
4. Schneier, B., Kelsey, J., Whiting, D., Wagner, D.,Hall, C., Ferguson, N. Performance Comparisons of the AES submissions. (1999)
5. Speed Comparison of Popular Crypto Algorithms. (n.d.). Retrieved April 17, 2022, from https://www.cryptopp.com/benchmarks.html
6. Blum, Lenore; Blum, Manuel; Shub, Michael (1983). "Comparison of Two Pseudo-Random Number Generators". Advances in Cryptology. Boston, MA: Springer US. doi:10.1007/978-1- 4757-0602-4_6.
7. Bernstein, Daniel (28 January 2008), ChaCha, a variant of Salsa20 (PDF), retrieved 21-11-2021
8. P. H. Tseng, M. H. Lee, Y. H. Lin, H. L. Lung, K. C. Wang, and C. Y. Lu, "ReRAM-Based Pseudo-True Random Number Generator with High Throughput and Unpredictability Characteristics," IEEE Trans. Electron Devices, vol. 68, no. 4, pp. 1593–1597, 2021.
9. C. E. C. Souza, D. P. B. Chaves, and C. Pimentel, "One-Dimensional Pseudo-Chaotic Sequences Based on the Discrete Arnold's Cat Map over Z3m," IEEE Trans. Circuits Syst. II Express Briefs, vol. 68, no. 1, pp. 491–495, 2021.
10. M. Jafari Barani, P. Ayubi, M. Yousefi Valandar, and B. Y. Irani, "A new Pseudo random number generator based on generalized Newton complex map with dynamic key," J. Inf. Secur. Appl., vol. 53, p. 102509, 2020.
11. P. Ayubi, S. Setayeshi, and A. M. Rahmani, "Deterministic chaos game: A new fractal based pseudo-random number generator and its cryptographic application," J. Inf. Secur. Appl., vol. 52, p. 102472, 2020.
12. A. Akgul, B. Gurevin, I. Pehlivan, M. Yildiz, M. C. Kutlu, and E. Guleryuz, "Development of microcomputer based mobile random number generator with an encryption application," Integration, vol. 81, no. December 2020, pp. 1–16, 2021.
